# How well do neurosurgeons predict survival in patients with high-grade glioma?

**DOI:** 10.1007/s10143-021-01613-2

**Published:** 2021-08-12

**Authors:** Lisa Millgård Sagberg, Asgeir S. Jakola, Ingerid Reinertsen, Ole Solheim

**Affiliations:** 1 Department of Public Health and Nursing, Norwegian University of Science and Technology, Trondheim, Norway; 2 Department of Neurosurgery, St Olavs University Hospital, Olav Kyrres gt 17, 7006 Trondheim, Norway; 3 Department of Neurosurgery, Sahlgrenska University Hospital, Gothenburg, Sweden; 4 Institute of Neuroscience and Physiology, University of Gothenburg, Sahlgrenska Academy, Gothenburg, Sweden; 5 Department of Circulation and Medical Imaging, Norwegian University of Science and Technology, Trondheim, Norway; 6 Department of Health Research, SINTEF Digital, Trondheim, Norway; 7 Department of Neuromedicine and Movement Science, Norwegian University of Science and Technology, Trondheim, Norway

**Keywords:** Glioma, Brain neoplasms, Surgery, Prognosis

## Abstract

Due to the lack of reliable prognostic tools, prognostication and surgical decisions largely rely on the neurosurgeons’ clinical prediction skills. The aim of this study was to assess the accuracy of neurosurgeons’ prediction of survival in patients with high-grade glioma and explore factors possibly associated with accurate predictions. In a prospective single-center study, 199 patients who underwent surgery for high-grade glioma were included. After surgery, the operating surgeon predicted the patient’s survival using an ordinal prediction scale. A survival curve was used to visualize actual survival in groups based on this scale, and the accuracy of clinical prediction was assessed by comparing predicted and actual survival. To investigate factors possibly associated with accurate estimation, a binary logistic regression analysis was performed. The surgeons were able to differentiate between patients with different lengths of survival, and median survival fell within the predicted range in all groups with predicted survival < 24 months. In the group with predicted survival > 24 months, median survival was shorter than predicted. The overall accuracy of surgeons’ survival estimates was 41%, and over- and underestimations were done in 34% and 26%, respectively. Consultants were 3.4 times more likely to accurately predict survival compared to residents (*p* = 0.006). Our findings demonstrate that although especially experienced neurosurgeons have rather good predictive abilities when estimating survival in patients with high-grade glioma on the group level, they often miss on the individual level. Future prognostic tools should aim to beat the presented clinical prediction skills.

## Introduction

Surgery is a cornerstone in the treatment of malignant brain tumors, and due to the incurable disease, it is crucial to balance the benefits against the risks. Due to the lack of reliable prognostic tools, both prognostication and surgical decisions largely rely on the neurosurgeons’ clinical prediction skills. However, clinical prediction is a difficult task, and the neurosurgeons’ predictive abilities are not yet much explored.

In a previous study, we demonstrated that neurosurgeons are overly optimistic when it comes to postoperative functional levels at 30 days in patients undergoing surgical resection for intracranial tumors [1]. Another study has found neurosurgeons to be overoptimistic regarding survival in patients with metastatic brain tumors undergoing radiosurgery [2]. In general, overpredictions of life expectancy are common in cancer patients [3–6], but most studies have focused on palliative oncologists and patients with terminal disease and short life expectancy.

In neurosurgery, knowledge about clinical prediction abilities is important since predictions may have large implications. Inaccurate predictions may lead to nihilistic or overly aggressive surgical strategies that are neither beneficial to the patients nor cost-effective. Also, it may affect the informed decision-making process and lead to unrealistic expectations for the patients.

In this prospective study, we aimed to assess the accuracy of the operating neurosurgeons’ prediction of survival in patients undergoing surgery for high-grade glioma. We also sought to explore factors possibly associated with accurate predictions.

## Material and methods

### Study design and study population

In this prospective study, high-grade glioma patients ≥ 18 years that were scheduled for surgical resections or diagnostic biopsies at the Neurosurgical Department, St. Olavs Hospital, Trondheim University Hospital, Norway, between September 2011 and December 2015 were eligible for inclusion. This department serves a population of about 750,000 inhabitants as the single neurosurgical department in one of Norway’s four geographical health regions. The tumors were histopathologically classified by a neuropathologist based on the 2007 WHO classification (molecular biomarkers not included) [7]. 

### Data collection and variables

Immediately after surgery, the operating surgeon predicted the patient’s survival on a questionnaire, using an ordinal scale (< 3 months, 3–6 months, 6–12 months, 12–18 months, 18–24 months, > 24 months). The surgeon also rated the patient’s preoperative functional status using Karnofsky Performance Status (KPS) scale at the same time. The actual survival time was calculated from time of surgery to death (end of follow-up = December 31, 2020).

Baseline and treatment data including new or worsened language deficits or motor deficits at discharge were collected from electronic medical records by one of three study nurses as part of a larger project. Charlson Comorbidity Index (CCI) [8] was used to classify comorbidity. Postoperative complications were registered according to the Landriel classification system[9]. Preoperative tumor volumes were semi-automatically segmented using the software 3D Slicer version 4.3.1–4.11 (3D Slicer, Boston, MA) or Brain Voyager™ QX version 1.2 (Brain Innovation, Maastricht, the Netherlands). In both software packages, T1-weighted contrast-enhanced images were used in contrast-enhancing lesions, and the tumor was defined as the volume of pathological contrast enhancement and necrotic tissue within the contrast-enhancing borders. Fluid attenuation inversion recovery (FLAIR) images were used in non-enhancing lesions. Eloquence was graded as suggested by Sawaya et al. [10].

### Surgical procedures and adjuvant treatment

Preoperative 3D MRI investigations were performed in most of the patients < 72 h before surgery by using a 1.5 or 3 Tesla MRI scanner and were supplemented with functional MRI and/or diffusion tensor imaging in the assumed most eloquent lesions. Surgical strategies were discussed in preoperative clinical meetings, and the operating surgeon informed the patients about the potential risks and benefits of surgery in preoperative consultations. Operating surgeons were either consultants or residents, and 15 different surgeons performed the procedures in the study period. Some of the surgeons were residents early in the period, and later became consultants. The patients either underwent craniotomies and tumor resections, or diagnostic biopsies only. All operations were performed under general anesthesia, and a neuronavigation system with 3D preoperative MRI and updated intraoperative 3D ultrasound volumes was used as needed [11]. During surgical resections, a frozen section was routinely sent to histopathological examination. After surgery, patients were referred to the oncological department for radiotherapy or chemotherapy according to protocols [12].

### Ethics and approvals

The study is based on informed consent from all participating patients. Data collection was approved by the Regional Ethical Committee for Health Region Mid-Norway (REC-number 2011/974) and adhered to guidelines of the Helsinki Declaration.

### Statistical analyses

All analyses were done using SPSS Statistics version 27.0. Q-Q plots and Shapiro–Wilk’s tests were used to test for normal distribution of data. Means ± SD were presented if data were normally distributed, while medians and interquartile range were presented if data were skewed. A Kaplan–Meier survival curve was used to visualize actual survival in groups based on the predicted survival scale, and the accuracy of clinical prediction was assessed by comparing predicted and actual survival. Due to few patients with predicted survival 18–24 months (*n* = 14), the predicted survival groups 12–18 months and 18–24 months were merged into one group (12–24 months). Predictions were considered accurate when actual survival fell within the predicted range. Overestimations were defined as actual survival shorter than predicted, and underestimations as actual survival longer than predicted. To investigate factors possibly associated with accurate estimation, a binary logistic regression analysis was performed. Only variables with a statistical trend in univariable analyses were included in the final multivariable model (*p* < 0.10), and variables with < 15 cases were excluded. Univariate Cox proportional hazards regression was used to calculate hazard ratios in a subgroup of patients, and the proportionality assumption was checked using log minus log plots. Statistical significance level was set at *p* ≤ 0.05.

## Results

**Fig. 1:**
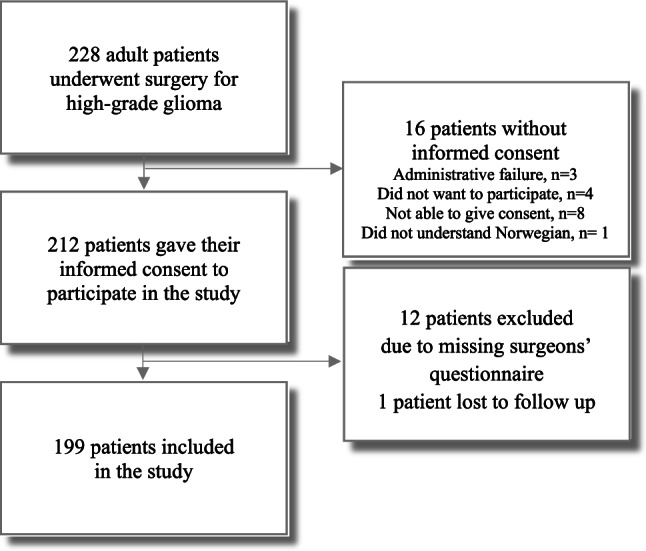
Flow chart of the inclusion process

A flow chart of the inclusion process is presented in Fig. 1. In total, 199 of 228 eligible patients with suspected and later confirmed high-grade glioma were included in the study.

Patient and treatment characteristics for all patients and for each predicted survival group are presented in Table 1. The median age of all patients was 60 years, and 37% were female. Most patients were functionally independent, with a preoperative KPS score of ≥ 70 (*n* = 151, 76%). At time of diagnosis, patients presented a range of neurological symptoms, where the most common were cognitive impairment (39%), headache (32%), balance/coordination problems (32%), epileptic seizures (29%), and language problems (27%). In total, 63% were primary operations and 82% of the operations were resections. The most common histopathology was glioblastoma (80%). A consultant was the primary operating surgeon in 76% of the procedures. As also demonstrated in the table, several patient and treatment characteristics were unevenly distributed between predicted survival groups. The patient’s age was the lowest in the group with predicted survival of > 24 months, and the KPS was gradually higher than the longer life expectancy. Furthermore, residents more often predicted shorter survival times, and extent of resection was lower in patients with short life expectancy.

**Table 1:** Patient and treatment characteristics

	All patients	Predicted survival groups			
	*n* = 199	3–6 mo *n* = 20	6–12 mo *n* = 84	12–24 mo *n* = 53	> 24 mo *n* = 42
Age in years, median (IQR*)	60 (16)	63 (16)	64 (14)	62 (16)	47 (20)
Female gender, *n* (%)	74 (37)	9 (45)	38 (45)	13 (25)	14 (33)
Preoperative Karnofsky Performance status, *n* (%)					
≥ 80	107 (54)	3 (15)	31 (37)	36 (68)	37 (88)
70	44 (22)	7 (35)	20 (24)	13 (25)	4 (10)
≤ 60	48 (24)	10 (50)	33 (39)	4 (8)	1 (2)
Charlson Comorbidity Index ≥ 2, *n* (%)	10 (5)	1 (5)	4 (5)	2 (4)	3 (7)
Preoperative symptoms, *n* (%)					
Headache	63 (32)	8 (40)	33 (39)	19 (36)	3 (7)
Epileptic seizures	58 (29)	3 (15)	14 (17)	19 (36)	22 (52)
Cognitive impairment	77 (39)	9 (45)	39 (46)	24 (45)	5 (12)
Nausea/vomiting	28 (14)	3 (15)	17 (20)	6 (11)	2 (5)
Balance/coordination problems	64 (32)	10 (50)	36 (43)	14 (26)	4 (10)
Visual problems	14 (7)	1 (5)	6 (7)	6 (11)	1 (2)
Language problems	53 (27)	7 (35)	30 (36)	11 (21)	5 (12)
Cranial nerve deficits	2 (1)	0 (0)	2 (2)	0 (0)	0 (0)
Motor symptoms	41 (21)	5 (25)	27 (32)	7 (13)	2 (5)
Preoperative tumor volume**, cm^3^, median (IQR*)	20.9 (38.7)	26.3 (24.8)	24.9 (47.5)	19.8 (33.1)	14.3 (27.7)
Eloquent tumor location (Sawaya grade 3), *n* (%)	89 (45)	13 (65)	40 (48)	15 (28)	21 (50)
Multifocal tumor, *n* (%)	42 (21)	3 (15)	22 (26)	11 (21)	6 (14)
Operating surgeon, *n* (%)					
Consultant	151 (76)	11 (55)	59 (70)	43 (81)	38 (91)
Resident	48 (24)	9 (45)	25 (30)	10 (19)	4 (10)
Primary operation, *n* (%)	126 (63)	11 (55)	53 (61)	45 (85)	17 (41)
Extent of resection,*** n (%)					
Gross total resection (100%)	52 (26)	1 (5)	14 (17)	22 (42)	15 (36)
Subtotal resection (1–99%)	109 (55)	11 (55)	49 (60)	23 (43)	26 (62)
Biopsy only	36 (18)	8 (40)	19 (23)	8 (15)	1 (2)
Histopathology, *n* (%)					
Glioblastoma (WHO-grade IV)	160 (80)	18 (90)	76 (91)	48 (91)	18 (43)
WHO-grade III/unspecified high-grade glioma	39 (20)	2 (10)	8 (10)	5 (9)	24 (57)

^*^IQR interquartile range

^**^ Missing preop. 3D MRI, *n* = 2

^***^Missing postop. MRI, *n* = 2 (resections)

**Fig. 2:**
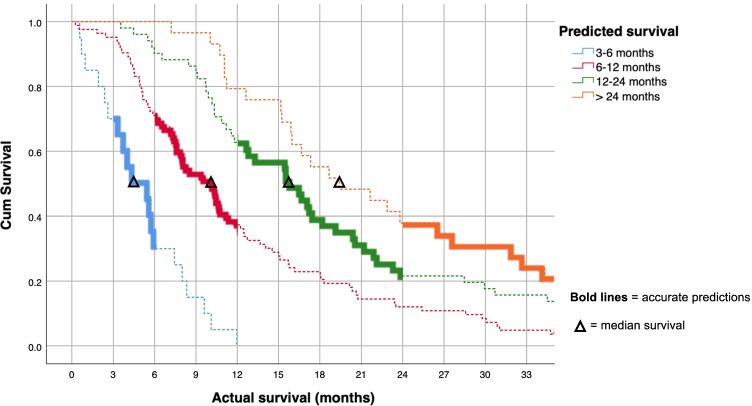
Kaplan–Meier curves for different clinical prediction groups

Differences between actual survival and predicted survival are presented in a Kaplan–Meier survival curve where the lines represent groups based on the predicted survival scale. Bold lines indicate accurate predictions (Fig. 2). As seen, patients with predicted survival 3–6 months had the shortest actual survival (median 4.5 months). Furthermore, patients with predicted survival 6–12 months had a median survival of 10.2 months, patients with predicted survival 12–24 months had a median survival of 15.7 months, and patients with predicted survival > 24 months had the longest actual survival (median 19.4 months). The differences across prediction groups were statistically significant (log rank *p* < 0.001). The overall accuracy was 41%, while the surgeon overestimated and underestimated the survival time in 34% and 26% of the patients, respectively. No patients had predicted survival < 3 months, but 10 patients (5%) died within 3 months of surgery. In total, 15 patients were still alive at end of follow-up, but all of them survived > 24 months.

In a binary logistic regression model, we investigated pre- and postoperative factors possibly associated with accurate prediction (Table 2). Using the Box-Tidwell procedure, all continuous independent variables were found to be linearly related to the logit of the dependent variable. We found no evidence of multicollinearity as assessed by tolerance values > 0.1. First, possible predictor variables were tested in univariable analyses, where gender, preoperative KPS, preoperative nausea/vomiting, preoperative balance/coordination problems, tumor edema, multifocal tumor location, type of surgeon, and histopathology had *p* < 0.10. When including these variables in the final multivariable model, only consultant as primary surgeon remained as a statistically significant factor for accurate prediction. Consultants were 3.4 times more likely to accurately predict survival compared to residents (*p* = 0.006). There were three standardized residuals, but all had a value of < 2.5 standard deviations and was kept in the analyses. The model was statistically significant, *χ*^2^ = 29.992 (*p* < 0.001), and correctly identified 68.5% of cases. The concordance index, a measure of the predictive accuracy of the model, was 0.72.

**Table 2:** Factors possibly associated with accurate prediction

	Univariable analyses		Multivariable analysis	
	Odds ratio (95% CI)	*p*-value	Odds ratio (95% CI)	*p*-value
Age in years (continuous)	0.98 (0.96–1.00)	0.113		
Female patient gender, y/*n*	0.52 (0.29–0.95)	0.035	0.53 (0.27–1.04)	0.061
Preoperative KPS* (ordinal)	1.02 (1.00–1.04)	0.024	1.00 (0.97–1.02)	0.786
Preoperative symptoms				
Headache, y/*n*	1.04 (0.56–1.90)	0.912		
Epileptic seizures, y/*n*	1.27 (0.69–2.36)	0.448		
Cognitive impairment, y/*n*	0.81 (0.45–1.46)	0.488		
Nausea/vomiting, y/n	0.44 (0.18–1.08)	0.074	0.46 (0.17–1.22)	0.119
Balance/coordination problems, y/*n*	0.50 (0.26–0.94)	0.031	0.74 (0.36–1.5142)	0.406
Language problems, y/*n*	1.29 (0.69–2.44)	0.429		
Motor symptoms, y/*n*	1.18 (0.59–2.36)	0.640		
Preoperative tumor volume, cm^3^ (continuous)	1.00 (0.99–1.01)	0.939		
Maximum peritumoral edema, mm (continuous)	0.98 (0.96–1.00)	0.081	0.99 (0.97–1.02)	0.671
Tumor lateralization				
Right	Reference			
Left	0.94 (0.38–2.32)	0.898		
Bilateral/midline	1.32 (0.53–3.25)	0.543		
Eloquent tumor location (Sawaya grade 3), y/*n*	1.16 (0.66–2.05)	0.607		
Multifocal tumor, y/*n*	0.38 (0.17–0.82)	0.014	0.49 (0.21–1.13)	0.094
Tumor location				
Frontal	Reference			
Temporal	1.05 (0.35–3.12)	0.936		
Multiple lobes	1.05 (0.34–3.22)	0.924		
Other**	0.43 (0.15–1.18)	0.102		
Consultant as primary surgeon, y/*n*	3.95 (1.79–8.72)	< 0.001	3.38 (1.42–8.07)	0.006
Primary operation, y/*n*	0.82 (0.45–1.46)	0.494		
Extent of resection (ordinal)	1.39 (0.90–2.16)	0.142		
WHO-grade III/unspecified HGG***, y/*n*	2.22 (1.09–4.50)	0.028	2.27 (0.97–5.32)	0.059
Grade II–IV complications, y/*n*	1.14 (0.47–2.74)	0.773		
New or worsened language or motor deficits at discharge, y/*n*	1.50 (0.74–3.04)	0.258		

^*^KPS Karnofsky Performance status

^**^Parietal, occipital, or deep cerebral

^***^HGG High-grade glioma

**Fig. 3:**
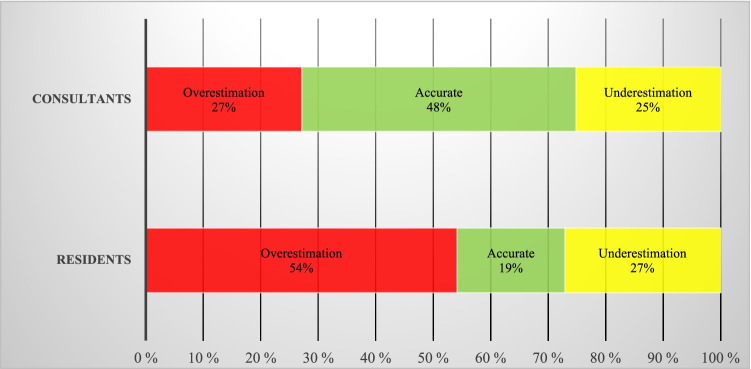
Accuracy based on surgeon’s experience

The surgeons’ prediction abilities were further explored in post hoc analyses. In Fig. 3, the accuracy based on clinical experience is presented. As seen, consultants had accurate predictions in 47% of the cases, and overestimated and underestimated survival in 27% and 25%, respectively. Residents had accurate predictions in only 19% of the cases, and overestimated survival in 54%. In patients with WHO grade III gliomas and unspecified high-grade gliomas, the predictive accuracy was 56%, and underestimations were made in only three cases (8%).

To compare the surgeons’ survival predictions to a prognostic score, a subgroup with primary glioblastoma only (*n* = 105) was also divided into prognostic risk groups based on a recursive partitioning analysis (RPA) [13]. Only 4 of 105 patients were considered to have low or low-moderate risk (i.e., RPA group 1 or 2). Using univariate Cox proportional hazards regression analyses, the hazard ratio was 1.80 (95% CI 1.32–2.46, *p* < 0.001) for the surgeons’ clinical prediction, and 1.93 (95% CI 1.34–2.79, *p* < 0.001) for the RPA groups.

## Discussion

In this prospective study, we assessed the accuracy of operating neurosurgeons’ prediction of survival in a population-based high-grade glioma cohort. Using an ordinal scale, we found that the surgeons were able to differentiate between patients with different length of survival, and the median survival fell within the predicted range in all groups with predicted survival < 24 months. In the group with predicted survival > 24 months, the median survival was shorter than predicted, indicating that long-term survival may be more difficult to estimate. The overall accuracy of surgeons’ survival estimates was still only 41%, and both over- and underestimations were common. Consultants were more than three times more likely to predict survival accurately compared to residents. Our findings demonstrate that although especially experienced neurosurgeons have rather good predictive abilities on the group level, they often miss on the individual level. A prognostic RPA score did not provide much better discrimination than the surgeons’ estimate.

To our knowledge, this is the first study that provides insight into neurosurgeons’ prediction of survival in patients with glioma. Other studies of survival prediction have almost exclusively been done in advanced cancer patients with short life expectancy. However, one study have examined a group of cancer specialists’ (including six neurosurgeons) ability to predict survival in patients with brain metastases undergoing radiosurgery [2]. In these studies, either continuous, probabilistic, or categorical prediction scales have been used, in different settings, with different definitions of accuracy, and different cutoffs, making both interpretations and comparisons difficult [3, 6, 14]. Accuracy rates from 23 to 85% have been reported, with a tendency towards overestimations, but a systematic review summarizes that clinical predictions of survival are still related to actual survival and thus have discriminatory ability [3]. This is in line with our findings where the surgeons were able to differentiate between groups with different lengths of survival, although their accuracies were still limited on the individual level.

Survival predictions are often reported to be more accurate towards the end of the patients’ life, [3, 6], and this so-called horizon-effect may explain why overestimations were most common in the group with the longest expected survival. In the neurosurgical setting, the time frame is also usually longer than in palliative oncology, and neurosurgeons meet their glioma patients early in the disease when they still are in fairly good condition. However, since our ordinal prediction scale was unevenly distributed, the probability to fall within the predicted range was larger when the time interval was longer. In addition, underestimations were impossible in the group with predicted survival of > 24 months. Consequently, the accuracy rate was highest in this group.

Clinical prediction of survival is not based entirely on simple intuition or so-called gut feeling. The neurosurgeons are likely to subjectively integrate a range of known prognostic factors when making estimates, including patients’ age, KPS, mental status, comorbidity, MRI-findings, preliminary histopathological findings from frozen section, and estimated residual tumor volume. In the present study, especially the factors age and KPS seemed to influence the surgeons’ estimates largely. Since knowledge of prognostic factors is important when making survival estimates, it is not surprising that we found consultants to have more accurate predictions than residents. They also had a more even distribution between over- and underestimation. In contrast, the residents had accurate predictions in only 19% of their cases, and overestimated survival in the majority of patients. Since consultants and residents perform different operations, this variation may be caused by selection bias. However, several other studies have also reported an association between accurate survival estimates and clinical experience [15, 16]. Presumably, training in prognostication can improve the accuracy of clinical prediction. In addition, an extended knowledge of the total disease trajectory is important. This could explain why neurosurgeons are found to be more optimistic and less accurate to predict survival in patients with brain metastases who died within 1 year compared to medical/neurooncologists [2].

As demonstrated in the present study, using a prognostic score do not necessarily provide much better discrimination between risk groups than simple clinical prediction. A number of prognostic scores based on multiple factors independently associated with survival in high-grade gliomas have been developed [13, 17–20]. So far, they are only able to predict short, medium, or long survival, and give no accurate estimates. Furthermore, all factors to be used for scoring are not always known at the time of surgery. Thus, the surgeon’s predictive abilities are still the most important and decisive factor in many cases. However, using clinical prediction of survival as an adjunct to improve the accuracy of prognostic factors or scores is recommended in advanced cancer patients [21], and perhaps this might be useful also in surgical glioma patients. Still, it remains to be demonstrated that prognostic tools perform better than the presented clinical estimates.

To move away from intuitive clinical prediction skills and integrate more data from the patient at hand as well as previous patients, several survival prediction methods based on artificial intelligence have also been developed over the last few years. Many of these have been proposed in the context of the yearly BRATS challenge which since 2017 includes a task on prediction of overall survival for glioma patients with gross total resection based on preoperative MRIs and age [22]. So far, these efforts have not reached clinically relevant accuracy, probably due to small training sets and limited number of clinical variables available. In addition, the classification of patients into short, medium, and long survival is relatively coarse and not necessarily optimal from a clinical point of view. With more patients and more relevant clinical variables available, there may be a potential for more accurate predictions at the individual level in the future.

### Strengths and limitations

The strength of the present study is the prospective population-based cohort, increasing the generalizability of findings. A possible limitation is that our results depend much on the chosen cutoffs in the ordinal prediction scale. In addition, our data collection started before molecular markers were integrated in the WHO classification.

## Conclusions

Our findings demonstrate that operating neurosurgeons exhibit rather good predictive abilities when estimating survival in patients with high-grade glioma on the group level, but they often miss on the individual level. The surgeons’ predictive abilities seem to improve with clinical experience. Future prognostic tools should aim to beat the presented clinical prediction of survival.

## Data Availability

The data that support the findings are available from the corresponding author, upon reasonable request.

## Code availability

N/A.

## References

[1] Sagberg LM, Drewes C, Jakola AS, Solheim O (2017). Accuracy of operating neurosurgeons’ prediction of functional levels after intracranial tumor surgery. J Neurosurg.

[2] Kondziolka D, Parry PV, Lunsford LD, Kano H, Flickinger JC, Rakfal S, Arai Y, Loeffler JS, Rush S, Knisely JPS, Sheehan J, Friedman W, Tarhini AA, Francis L, Lieberman F, Ahluwalia MS, Linskey ME, McDermott M, Sperduto P, Stupp R (2014). The accuracy of predicting survival in individual patients with cancer. J Neurosurg.

[3] Glare P, Virik K, Jones M, Hudson M, Eychmuller S, Simes J, Christakis N (2003). A systematic review of physicians’ survival predictions in terminally ill cancer patients. BMJ.

[4] Clement-Duchene C, Carnin C, Guillemin F, Martinet Y (2010). How accurate are physicians in the prediction of patient survival in advanced lung cancer?. Oncologist.

[5] Cheon S, Agarwal A, Popovic M, Milakovic M, Lam M, Fu W, DiGiovanni J, Lam H, Lechner B, Pulenzas N, Chow R, Chow E (2016). The accuracy of clinicians’ predictions of survival in advanced cancer: a review. Ann Palliat Med.

[6] Chow E, Harth T, Hruby G, Finkelstein J, Wu J, Danjoux C (2001). How accurate are physicians’ clinical predictions of survival and the available prognostic tools in estimating survival times in terminally ill cancer patients? A systematic review. Clin Oncol (R Coll Radiol).

[7] Louis DN, Ohgaki H, Wiestler OD, Cavenee WK, Burger PC, Jouvet A (2007). The 2007 WHO classification of tumours of the central nervous system. Acta Neuropathol.

[8] Charlson ME, Pompei P, Ales KL, MacKenzie CR (1987). A new method of classifying prognostic comorbidity in longitudinal studies: development and validation. J Chronic Dis.

[9] Landriel Ibanez FA, Hem S, Ajler P, Vecchi E, Ciraolo C, Baccanelli M, al. e,  (2011). A new classification of complications in neurosurgery. World Neurosurg..

[10] Sawaya R, Hammoud M, Schoppa D, Hess KR, Wu SZ, Shi WM, Wildrick DM (1998). Neurosurgical outcomes in a modern series of 400 craniotomies for treatment of parenchymal tumors. Neurosurgery..

[11] Gronningsaeter A, Kleven A, Ommedal S, Aarseth TE, Lie T, Lindseth F (2000). SonoWand, an ultrasound-based neuronavigation system. Neurosurgery..

[12] Weller M, van den Bent M, Tonn JC, Stupp R, Preusser M, Cohen-Jonathan-Moyal E, Henriksson R, Le Rhun E, Balana C, Chinot O, Bendszus M, Reijneveld JC, Dhermain F, French P, Marosi C, Watts C, Oberg I, Pilkington G, Baumert BG, Taphoorn MJB, Hegi M, Westphal M, Reifenberger G, Soffietti R, Wick W (2017). European Association for Neuro-Oncology (EANO) guideline on the diagnosis and treatment of adult astrocytic and oligodendroglial gliomas. Lancet Oncol.

[13] Lamborn KR, Chang SM, Prados MD (2004). Prognostic factors for survival of patients with glioblastoma: recursive partitioning analysis. Neuro Oncol.

[14] White N, Reid F, Harris A, Harries P, Stone P (2016). A systematic review of predictions of survival in palliative care: how accurate are clinicians and who are the experts?. PLoS ONE.

[15] Taniyama TK, Hashimoto K, Katsumata N, Hirakawa A, Yonemori K, Yunokawa M, Shimizu C, Tamura K, Ando M, Fujiwara Y (2014). Can oncologists predict survival for patients with progressive disease after standard chemotherapies?. Curr Oncol.

[16] Christakis NA, Lamont EB (2000). Extent and determinants of error in physicians’ prognoses in terminally ill patients: prospective cohort study. West J Med.

[17] Chaichana K, Parker S, Olivi A, Quinones-Hinojosa A (2010). A proposed classification system that projects outcomes based on preoperative variables for adult patients with glioblastoma multiforme. J Neurosurg.

[18] Marko NF, Weil RJ, Schroeder JL, Lang FF, Suki D, Sawaya RE (2014). Extent of resection of glioblastoma revisited: personalized survival modeling facilitates more accurate survival prediction and supports a maximum-safe-resection approach to surgery. J Clinical Oncol..

[19] Li J, Wang M, Won M, Shaw EG, Coughlin C, Curran WJ, Mehta MP (2011). Validation and simplification of the Radiation Therapy Oncology Group recursive partitioning analysis classification for glioblastoma. Int J Radiat Oncol Biol Phys.

[20] Curran WJ, Scott CB, Horton J, Nelson JS, Weinstein AS, Fischbach AJ, Chang CH, Rotman M, Asbell SO, Krisch RE (1993). Recursive partitioning analysis of prognostic factors in three Radiation Therapy Oncology Group malignant glioma trials. J Natl Cancer Inst.

[21] Maltoni M, Caraceni A, Brunelli C, Broeckaert B, Christakis N, Eychmueller S, Glare P, Nabal M, Vigano A, Larkin P, De Conno F, Hanks G, Kaasa S, Steering Committee of the European Association for Palliative C (2005). Prognostic factors in advanced cancer patients: evidence-based clinical recommendations–a study by the Steering Committee of the European Association for Palliative Care. J Clin Oncol.

[22] Bakas S, Reyes M, Jakab A, Mauer S, Rempfler M, Crimi A et al (2018) Identifying the Best Machine Learning Algorithms for Brain Tumor Segmentation, Progression Assessment, and Overall Survival Prediction in the BRATS Challenge. arXiv:181102629

